# Basic Principles of the Validation for Good Laboratory Practice Institutes

**DOI:** 10.5487/TR.2009.25.1.001

**Published:** 2009-03-01

**Authors:** Kyu-Hyuk Cho, Jin-Sung Kim, Man-Soo Jeon, Kyuhong Lee, Moon-Koo Chung, Chang-Woo Song

**Affiliations:** 11 Division of Inhalation Toxicology, Korea Institute of Toxicology, Korea Research Institute of Chemical Technology, 1051 ShinJeong-dong, JeongEup-si, Jeollabuk-do, 580-185 Korea; 21 Department of Non-clinical Studies, Korea Institute of Toxicology, Korea Research Institute of Chemical Technology, DeaJeon, 305-343 Korea; 31 Korea GMP Academy Co., Ltd., Chungcheongbuk-do, 363-794 Korea

**Keywords:** Good laboratory practice, Validation, Qualification, Calibration

## Abstract

Validation specifies and coordinates all relevant activities to ensure compliance with good laboratory practices (GLP) according to suitable international standards. This includes validation activities of past, present and future for the best possible actions to ensure the integrity of non-clinical laboratory data. Recently, validation has become increasingly important, not only in good manufacturing practice (GMP) institutions but also in GLP facilities. In accordance with the guideline for GLP regulations, all equipments used to generate, measure, or assess data should undergo validation to ensure that this equipment is of appropriate design and capacity and that it will consistently function as intended. Therefore, the implantation of validation processes is considered to be an essential step in a global institution. This review describes the procedures and documentations required for validation of GLP. It introduces basic elements such as the validation master plan, risk assessment, gap analysis, design qualification, installation qualification, operational qualification, performance qualification, calibration, traceability, and revalidation.

## INTRODUCTION

Validation is defined to verify the confidence, integrity, and traceability of data which are generated in the entire manufacturing process of drugs and pharmaceuticals as well as in non-clinical laboratory study. Establishing documented evidence that provides a high degree of assurance that a specific process (*e.g.*, operation, process, utilities, study and system) will consistently produce a product that meets predetermined specifications and quality (FDA, 1987). Some management of drug and pharmaceutical supplies and non-clinical laboratory studies are related directly or indirectly with people's health. Therefore, facilities that engage in non-clinical laboratory studies must comply with quality-control validation.

Guidelines for the manufacture process of pharmaceutical supplies and for non-clinical laboratory studies include GMP (good manufacturing practice) and GLP (good laboratories practice) (EPA, 1989; OECD, 1997; US FDA, 2003; KFDA, 2005, 2007; MIHWAF, 2008). More extensive guidelines for quality-control regulation are provided in *A WHO Guide to Good Manufacturing Practice (GMP) Requirements, Part 2: Validation* (WHO, 1996), *Guideline on General Principles of Process Validation* (FDA, 1987), *Guide to Good Manufacturing Practice, Annex 15: Qualification and Validation* (EU GMP, 2003), and *Principle of Validation for Drug and Pharmaceutical* (KFDA, 2008).

The guidelines for non-clinical laboratory studies clearly describe that equipments should be inspected, cleaned, and maintained with an approved protocol. Instruments used for the generation, measurement, or assessment of data should also be tested, calibrated, and standardized with an approved protocol (U.S FDA 58.61 and 63, EPA 792.61, OECD Section II 4.1 and 4.2, and II. KFDA 4.(1, 2). And US FDA 58.81, EPA

792.81 OECD sections II 7.4 and II. and KFDA 7.5(2) state that facility inspection, cleaning, maintenance, testing (validation), calibration, and standardization of equipment should occur and that appropriate remedial action should be taken in the event of equipment failure or malfunction (EPA, 1989; OECD, 1997; US FDA, 2003; KFDA, 2005; Weinberg, 2007; Yoshihito, 1985; Yu *et al.*, 1999, 2000). According to the last revision of Korea Good Laboratory Practice interpretation text, computer systems that contain measuring devices and control the environment and disinfection of animal cages, racks, and accessories should be checked for integrity and reproducibility of results (KFDA, 2008).

Whereas validation is internationally required to verify the result of non-clinical laboratory study data, which can influence people's health, validation execution of GLP laboratory institutions are not sufficient in most of Korea. In this review, the basic techniques for consistent, smooth validation are discussed.

## APPROACH METHODOLOGY

***Classification and personnel.*** Validation is classified according to the working process: (a) Process validation guarantees a reproducible process to follow a pre-specified rule for suitable and documented results; (b) Method validation verifies that any errors (deviations) in the analytical method for a given product (e.g., a pharmaceutical product) do not exceed a set measurement criteria; (c) Computer systems validation verifies the logic of computer programs as well as the secure management and storage of documents; (d) Cleaning validation is performed to prevent cross contamination; (e) Manufacture utilities system validation verifies the management system that controls air and water for injection, process management, monitoring, and common appliances; (f) Equipment validation ensures that equipment made by a manufacture is working according to its intended use and role (Gibson and Powell-Evans, 1998; KPTA, 2000; EU 2003).

Validation can also be classified by timing: (a) Prospective validation is the preliminary inspection of risk factors of a process with the percentage integrity of each item to measure; (b) Concurrent validation deals with a specific condition, such as process transfer or defined process; (c) Revalidation is gauged within state interval or basic period, or quality deterioration of equipment; (d) Retrospective validation is demonstrated after pharmaceutical product has reached the market; (e) Change control is performed to prevent machine deterioration, part change that can affect results and measurements are changed if they deviate from normal (FDA, 1987; KPTA, 2000).

Generally, the facility for non-clinical laboratory studies has a validation structure similar to that shown in Fig. 1. This structure includes the validation committee, validation manager, validation specialist (or validation study director), equipment, and the qualification

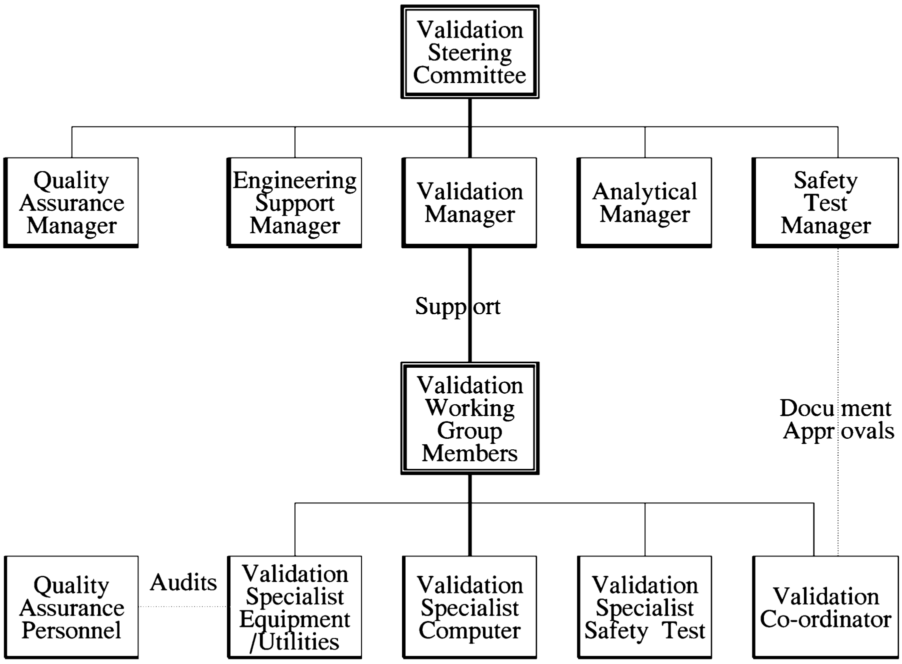

**Fig. 1.** The Team and Working Party Structure (Modified from Gibson and Powell-Evans, 1998).

team needed to execution the validation: (a) The validation committee makes decisions about policy, documents, activity processes, and judges the validation result etc; (b) The validation manager, who is a part of the validation committee, controls the general progress, and creates the validation master plan; (c) The validation specialist, also called a validation study director, performs the validation process following the validation master plan;. (d) Validation team writes the protocol, perform the qualification, and submit the final report (Gibson and Powell-Evans, 1998).

***Basic components.*** When a validation specialist performs a routine validation, the following basic components are included: (a) Validation master plan includes the best verification policy that the integrity of all processes should be measured against; (b) Gap analysis estimates the difference between the present condition of the equipment and the future goal; (c) Risk analysis, includes finding the best method to reduce or avoid a given risk; (d) User requirement specifications comprise a purchase procedure, which must be written concretely and followed for the specification of equipment; (e) Function and design specification is the function and design of the equipment written in technical language by the manufacturer or vendor; (f) System requirement specification is the summary of the facility, an essential part that requires maintenance and a measurement method for an equipment or system.

During the equipment selection and installation process, it is necessary to perform the following validation steps: (a) vendor audit for selecting a creditable manufacturer/vendor to meet a user's requirements; (b) design qualification for evaluating a design for the user's requirements and specifications; (c) installation qualification for verifying that each evaluation item passed design specification and installation; (d) operational qualification for verifying that each operational procedure and test factor are able to satisfy the acceptance criteria through verification; (e) performance qualification for demonstrating that an instrument will function according to the operational specifications.

In addition, the following items must also be considered: process manual or standard operating procedure (SOP), record form, acceptance criteria for each piece of equipment, protocol written prior to testing, final report for the test results, change control after maintenance, rescue disaster, calibration method, cleaning, verification maintenance, security system, raw data, and an audit trail that has credible test results (Bedson and Sargent, 1996; WHO, 1996; Gibson and Powell-Evans, 1998; Huber, 1998; EU, 2003; PIC/S, 2003;

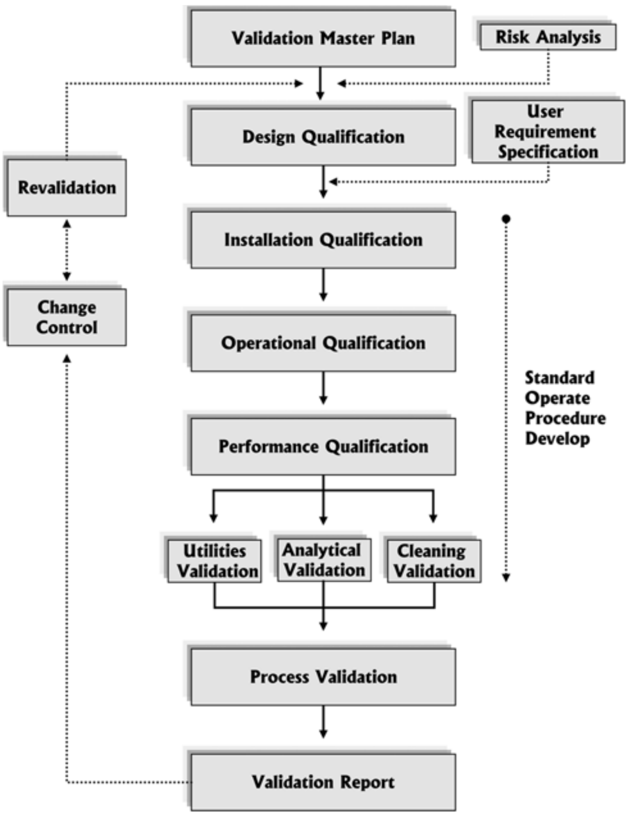

**Fig. 2.** Flow chart of validation process (Modified from Haider, 2002a, b).

KFDA, 2008).

As Fig. 2 shows the processes for accomplishing validation, each validation study director must establish a master plan with several necessary steps of validation components, which components for validation can be learned through technical experiences and education, and must lay out the validation study at the same time.

***Scope and process.***

***Validation master plan:*** The validation master plan is the concrete and overall document including the validation policy and validation execution plan in each GLP institution. This master plan is written for the object and scope of the validation, policy, and process method for each department, using validated equipment, validation target (equipment, computer system, software, utility, facility, testing method), regulation (GLP, GMP, GCP), and the special environment of each department or institution. In the validation master plan, the institution's name, role, place, and plan for the year are described. The responsibility and role for the characteristic jobs within the department (e.g., management, study director, validation steering committee, quality assurance unit, validation team, utility team, computational statistics teams) are defined in this plan (LabComplience, 2004; Bridges, 2005).

In addition, the following items are included in validation master plan: the floor plans for the building, the movement line for the building, the constitution for the utilities, local area network, air handling unit, water supply, sewage, validation overview, qualification content overview, set measure item, list of all equipment, evaluation of characterization, development of user specification, equipment purchase system, vendor list, selection tool, risk analysis, gap analysis, documentation, rescue of equipment, and references (WHO, 1996; Gibson and Powell-Evans, 1998; Haider, 2002a; LabComplience, 2004; Bridges, 2005; KFDA, 2008). After the validation master plan is reviewed and approved by the management or validation committee, a validation is carried out under the direction of the validation study director.

***Risk analysis:*** The purpose of risk analysis is to find risk factors in advance and to reduce or eliminate the factors that may affect a test result. The goal of risk analysis is to evaluate the factors on a scientific basis which include the requirements of regulatory administrations, integrity of the data, and GLP guidelines for beginning new studies, purchasing new equipment, writing user requirement specifications, performing vendor audits, evaluating functional requirement specifications, and performing change control. Although the items to be tested are the same, if the object of analysis is different, it is a fundamental rule that the analysis must be repeated for each object (Haider, 2002a; Yu, 2004; Bridges, 2005; Brockmeyer, 2006).

The risk analysis documents describe the role and responsibility of staff or department and the methods to process. The process of risk analysis is as follows. First, process identification is preformed, in which all systems are grouped by skill and equipment. Second, all GLP risks are identified. The integrity of results is checked, errors are measured, and potential risk factors are determined. Third, business risks are identified such as increase of repair charge, economic high possession and lack of data credit. Fourth, the expected risks are assessed. Fifth, it is evaluated that the severity of a risk is influenced on regulatory compliance and reputation. Sixth, the probability of detection is assessed, including how to find risks more quickly and how to decrease their influence. For example, the expectation and severity of risk impact can be used to determine the level of danger. The assigned risk classification and probability of detection can be used to classify the magnitude of danger (Yu, 2004). After the strategy for risk reduction is set up according to the performance step of risk analysis, the validation study director must apply this strategy to reduce risk.

***Gap analysis:*** The gap analysis contains a brief outline, observes GLP regulatory requirements, and is convenient for the process and validation strategy. It has the management of qualification execution plan, and makes a lot of facility maintenance validity (Bridges, 2005).

Gap analysis includes the role or responsibility of relevant personnel, feature of equipment to be evaluated, the model number, serial manufacture number, serial asset number, using purpose, location, document or SOP, number, equipment logs, date of last calibration, personnel training records, a validation certificate, system controls, data storage methods, risk type, the level of the responsible person, and validation specialist (validation study director) (Bridges, 2005).

Gap analysis offers the easy and convenient information for the validation process. The validation study director needs to cooperate with the manufacture or vendor, and to notify any peculiarity of the facility to them. The document for gap analysis is similar to those shown in Table 1.

***Design qualification:*** Design qualification is performed for design of equipment, evaluation of quality,

**Table 1.** Gap Analysis Record (Mortified from Huber, 2005)

| Description | Manufacturer | Model # | Serial # | Asset # | Use | Location | SOP # | Equip logs | Last Calib. | Training | Validation certificate | System control | Data storage method | Risk type & level | Part/ User |
|---|---|---|---|---|---|---|---|---|---|---|---|---|---|---|---|
| | | | | | | | | | | | | | | | |
| | | | | | | | | | | | | | | | |
| | | | | | | | | | | | | | | | |
| | | | | | | | | | | | | | | | |
| | | | | | | | | | | | | | | | |
| | | | | | | | | | | | | | | | |
| | | | | | | | | | | | | | | | |
| | | | | | | | | | | | | | | | |
| | | | | | | | | | | | | | | | |
| | | | | | | | | | | | | | | | |

quality and confidence level of manufacturer, and using purpose of validation specialist (or validation study director) for compatibility decision and equipment selection. The process of design qualification is as follows. First, simple specifications are documented prior to the purchase of equipments. Second, the manufacturer or vendor to supply equipments, which meet the user's specifications and functional requirements, should be selected. Third, the user's requirement specifications are presented and checked (Bedson and Sargent, 1996; Gibson and Powell-Evans, 1998; EU, 2003; PIC/S, 2003; KDFA, 2008).

Functional requirements, which the manufacturer or vendor presents to the user, must be checked as related contents: study object, facility manual, SOP, technique for operation, training, total sample volume, explanation of total sample volume, data acquisition, service, utilities, expendable supply, environmental conditions and ranges (e.g., humidity, temperature, vibration, dust), equipment maintenance method, calibration cycle (including contract that has a calibration cycle), item and cost, certificate, maintenance, warranty period, health, safety, and information about environmental issues.

If a user has the same or similar equipment, it is possible to perform a supplementary test using previous information. In order to confirm the details of the record (e.g., source code, development record of the equipment, method of using the equipment, calibration certification, test record, report of batch, certificate documentation for hardware and software, and certificate of qualification of the development-related engineer), it is necessary that the user visit the manufacturer's office or review the equipment document and the vendor inspection sheet (e.g, vendor audit checklist), as well as the certification that was inspected as escrow. After confirming the details, a user integrates all opinions and selects the manufacturer and intended equipment. A user must obtain accurate design and specifications, education, documents for quality management, quality assurance methods, all-embracing test plan for each system, application of change control, and automatic error control instrument and remedy (Bedson and Sargent, 1996; Gibson and Powell-Evans, 1998; EU, 2003; PIC/S, 2003; KFDA, 2008).

***Installation qualification:*** The installation qualification must be performed before the validation specialist uses the facility for a study. The installation qualification consists of equipment design features (e.g., materials of construction, clean ability) and installation conditions (e.g., wiring, utilities, functionality). The validation specialist must also check that an equipment and processes follow design specification. The process of installation qualification checks that the acquisition, equipment, and accessories follow the purchase specifications. The validation specialist must also check the installation environmental conditions (e.g., clean room requirements, temperature, and humidity) with the manufacture (Bedson and Sargent, 1996; Gibson and Powell-Evans, 1998; EU, 2003; PIC/S, 2003; KFDA, 2008).

After the validation specialist receives equipment, hardware, software, accessory and spare parts, he or she must check with the manufacture that they meet the purchase specifications. The validation specialist will check the operational manual for equipment installation, maintenance, training, calibration, safety training, equipment operation, software installation, and external damage of equipment by the validation specialist and the manufacturer, and then develops the draft SOP, which includes developing the calibration SOP for each apparatus, preventative maintenance items, and cleaning schedules (Bedson and Sargent, 1996; Gibson and Powell-Evans, 1998; EU, 2003; PIC/S, 2003; KFDA, 2008).

***Operational qualification:*** Operational qualification is performed to pass design qualification and installation qualification. The test items of facility must reproduce the perfect results in an installed environment. First of all, a user must learn the technical information about the equipments such as operation process, maintenance, safety items, cleaning and sterilization (Bedson and Sargent, 1996; Gibson and Powell-Evans, 1998; EU, 2003; PIC/S, 2003; KFDA, 2008).

The validation specialist develops a working SOP, checks that the facility follows the process of working SOP, and confirms that the equipment to check getting result indicating value of each alarms and interlock. This result must fulfill the test item and acceptance criteria, which set up system requirement specifications. When a high technical skill is required for an operational qualification of special equipment, the expert of manufacture should performs the qualification process (Bedson and Sargent, 1996; Gibson and Powell-Evans, 1998; EU, 2003; PIC/S, 2003; KFDA, 2008).

***Performance qualification:*** Performance qualification is performed for an operating facility that already has undergone design qualification, installation qualification, and operational qualification. This qualification is performed to ensure that the test items are reproducible with meeting the acceptance criteria, and show an intended use. This process is performed by the validation specialist, who demonstrates operation according to the procedure for operation and the SOP. The pro-

**Table 2.** Performance qualification report (Modified from LabCompliance, 2004)

| Instrument ID: | | | Study No.: | |
|---|---|---|---|---|
| Function 1: | Set point/Expected Value | Acceptance Limit | Actual | Decision |
| Parameter 1: | | | | □ Yes □ No |
| Parameter 2: | | | | □ Yes □ No |
| Deviations | | | | |
| Function 2: | Set point/Expected Value | Acceptance Limit | Actual | Decision |
| Parameter 1: | | | | □ Yes □ No |
| Parameter 2: | | | | □ Yes □ No |
| Deviations | | | | |
| Function 3: | Set point/Expected Value | Acceptance Limit | Actual | Decision |
| Parameter 1: | | | | □ Yes □ No |
| Parameter 2: | | | | □ Yes □ No |
| Deviations | | | | |

cess corrects for a deviation factor from each proof item, and establishes an intended use (Bedson and Sargent, 1996; Gibson and Powell-Evans, 1998; EU, 2003; PIC/S, 2003; KFDA, 2008).

The validation specialist checks that the test results are reproducible following accepted criteria. He or she checks the reproducibility of a test result more than three times, and should set up a plan to maintain the integrity of the equipment. Also, it is useful to test the results of limit value, low and high values, and worst case of equipment. The validation specialist conforms the SOP from the installation and operational qualifications (Bedson and Sargent, 1996; Gibson and Powell-Evans, 1998; EU, 2003; PIC/S, 2003; KFDA, 2008). The validation specialist combines the raw data, protocol, and report from each qualification, and prepares the final report. As example, the records for performance qualification results are shown in Table 2.

***Calibration and traceability:*** The sensitivity of the measuring instrument can affect the integrity of the equipment for producing data. Thus, ISO Guide 25, ISO 9000 Standards, and GLP guidelines require users to follow national and international standards that allow traceability for inspection and calibration. This process reflects the consistency of the measurement. The measuring instrument, which has been installed in a GLP facility, or the recording (e.g., pressure gauge, flow meter, thermometer, timer, balance, thermometer recorder, humidity recorder, wavelength, and controller system) are used to calibrate the equipment. Calibration must follow the calibration equipment list and calibration schedule, and be performed by a person who has mastered the calibration technique of an equipment. In particular, the calibration equipment list contains equipment, calibration cycle, form, and acceptance criteria. After calibration, it is necessary to attach the notification label, which specifies the calibration date, operation range, next calibration date, and calibration standard. As a result, the validation study director of GLP facility can check the calibration of the equipment in use (Bedson and Sargent, 1996, International Standard ISO/IEC 17025:2005, 2006).

***Revalidation:*** The facility or equipment, which have already been validated, must be revalidated if the validation specialist or validation study director changes the facility by intent (e.g., exceed the expire date, removal of equipment environment, change main part, change for function improvement, change of goal) (Gibson and Powell-Evans, 1998; Haider, 2002a; PIC/S, 2003; KFDA, 2008). The validation study director should perform revalidation after important changes are carried out, because these changes affect measurement accuracy. If any parts of a facility are changed, the validation study director needs to make the documents about the reason of the change, the distinguishing feature, and the advantages and disadvantages. The facility validation study director checks the verified document in the course of instrument development. If a software system is changed, the instrument user must consider compatibility with the existing software. If this is not done, the validation study director must upgrade the system verification to the manufacturer. After revalidation is completed, the facility must receive the appropriate tests and inspections. If a test and inspection needs to be re-verified, the validation study director compares a new result with an old result, and records the differences in the equipment log (Gibson and Powell-Evans, 1998; PIC/S, 2003;

KFDA, 2008).

## CONCLUDING REMARKS

Every GLP institutions, which performs non-clinical laboratory studies for the test articles such as chemicals or pharmaceutical or food additive, and submits them to regulatory affairs, should conduct the validation. The reasons that a validation performance is needed in GLP institution are as follows: (a) the GLP system is required by a regulatory affairs in order to product reliable documents through preparing a study plan and a study report under a systematic organized relationship of members and departments; (b) the performance of validation is required through revision of guideline and publications which help the actual application and manuals written by an expert (OECD, 1999; Weinberg, 2007; KFDA, 2008).

In particular, Weinberg (2007) reported that the use of US FDA GLP 58.63 (a) can be used to examine an item or system to determine its compliance with its specifications (US FDA, 2003; Weinberg, 2007). Thus, the validation of GLP institutions should be conducted without exception. Every organization can use a different approach to manage these problems, but they should all solve these problems through proper personnel education, or direct or indirect effects by validation (e.g., minimized noncompliance, reduced reworks, corporate image, corporate legal protection, satisfactory inspection, and utility cost reduction etc) (Gibson and Powell-Evans, 1998; Haider, 2002b). In conclusion, if the quality management system of every organization is established for validation as confident and verified control system gradually, it can be the motive to achieve the goal to become the GLP institution as international level.
